# Jellyfish Venom Peptides Targeting Human Potassium Channels Identified through Ligand Screening: Morphometric and Molecular Identification of the Species and Antibiotic Potential

**DOI:** 10.3390/md22080333

**Published:** 2024-07-24

**Authors:** Edirisinghe Arachchige Hashini Wasthala Edirisinghe, Buddhima Nirmani Athukorala, Minoli Perera, Bothunga Arachchige Shamali Dilhara Abeywardana, Polgahawattage Sachini Tarushika Sigera, Pasindu Eranga, Kavindu Dinuhara Theekshana, Mohamad Boudjelal, Rizwan Ali, Dinithi Champika Peiris

**Affiliations:** 1 Department of Zoology, Faculty of Applied Sciences, University of Sri Jayewardenepura, Nugegoda 10250, Sri Lanka; hashiniwe996@gmail.com (E.A.H.W.E.); dilharashamali@gmail.com (B.A.S.D.A.); pasinduerangaonline@gmail.com (P.E.);; 2 Genetics and Molecular Biology Unit, Faculty of Applied Sciences, University of Sri Jayewardenepura, Nugegoda 10250, Sri Lanka; 3 King Abdullah International Medical Research Center (KAIMRC), Medical Research Core Facility, and Platforms (MRCFP), King Saud bin Abdulaziz University for Health Sciences (KSAU-HS), Ministry of National Guard Health Affairs (MNGHA), Riyadh 11481, Saudi Arabiaaliri@mngha.med.sa (R.A.)

**Keywords:** *Catostylus* sp., venom peptides, antibiotic action, potassium channel inhibitors, molecular docking, life on earth

## Abstract

The relative lack of marine venom could be attributed to the difficulty in dealing with venomous marine animals. Moreover, the venom of marine animals consists of various bioactive molecules, many of which are proteins with unique properties. In this study, we investigated the potential toxic proteins of jellyfish collected for ligand screening to understand the mechanism of the toxic effects of jellyfish. Since taxonomic identification is problematic due to the lack of proper keys, we conducted morphological and molecular mitochondrial DNA sequencing from *COI* and *ITS* regions. The venom extract from nematocysts found along the bell margins was used for protein characterization using SDS-gel electrophoresis and nano-liquid chromatography-tandem mass spectrometry. Ligand screening for the most potent toxin and antibacterial and cytotoxicity assays were carried out. The phylogenetic tree showed distinct clustering from other *Catostylus* sp. The proteomic analysis revealed venom with many bioactive proteins. Only 13 venom proteins were identified with molecular weights ranging from 4318 to 184,923 Da, exhibiting the venom’s complexity. The overall toxin protein composition of *Catostylus* sp. venom was dominated by potassium channel toxin alpha-KTx. Molecular docking of toxin alpha-KTx 1.13 revealed high specificity towards the human voltage-gated potassium channel Kv3 with a high fitness score and a minimum energy barrier of −17.9 kcal/mol. Disc diffusion and cytotoxicity assays revealed potent antibacterial activity against *Klebsiella pneumoniae* with no cytotoxicity. Further studies on detailed characterization and therapeutic potentials are warranted.

## 1. Introduction

Jellyfish blooming is becoming a global problem, causing significant economic and health losses. Being members of the phylum Cnidaria, they have been identified as one of the oldest phyla of venomous animals [1]. The characteristic feature of the cnidarians is the nematocyst, an organelle located in the umbrella, tentacles, and oral arms [2]. The characteristics of nematocytes vary significantly among species. However, they retain an analogous mechanism involving the ejection of a coiled thread in a harpoon-like technique through the open operculum upon chemical and mechanical stimuli [2]. The thread then penetrates the tissues of prey or predator, injecting various toxins from the fluid matrix of nematocysts [3]. This event is a reliable biomechanical process in the animal kingdom, occurring within 3 m of physical contact at high velocity, creating a pressure of 7.7 GPa at the impact site [4].

Jellyfish produce venom to capture and digest prey, repel predators, and compete with other species [5]. Their venom comprises a complex mixture of components, from non-proteinaceous molecules to high molecular weight proteins and small peptides that have evolved over hundreds of millions of years [2]. Recently, attention has been paid to jellyfish venom due to its biological activities, including cytolytic [5], enzymatic [6], hemolytic [7], neurotoxic [8], cardiotoxic [6], insecticidal, and antimicrobial activities [9,10].

Among jellyfish, scyphozoans are frequently abundant and significant inhabitants in many ocean environments. They often come into accidental contact with humans, causing mild to severe envenomation [10]. *Catostylus* is one of the most common jellyfish genera found in the shallow coastal waters of the Indo-Pacific Ocean [11]. They exhibit seasonal variations in their envenomation ability, being more noxious during the breeding season [12]. Recurrent jellyfish blooms adversely affect a country’s economy as they impact fisheries [13] and tourist industries [14].

Though human encounters with jellyfish venom are becoming increasingly problematic in Sri Lanka, knowledge of the toxic effects of jellyfish is scarce. To fill this knowledge gap, we investigated the potential toxin protein components underlying the significant poisonous effects of the crude venom extracted from jellyfish from the West Coast of Sri Lanka. Here, we attempted to identify the sp. nematocysts. To accomplish this endeavor, the crude venom was extracted using ultrasonication on the isolated nematocysts rather than tissue homogenization, which we predicted would reduce the background level of extraneous proteins. The venom was then subjected to SDS-PAGE electrophoresis to separate high molecular weight proteins from the protein–protein complex. Studies were conducted to determine possible interactions between the venom neurotoxin and human potassium/sodium channels. We extracted the crude nematocyst venom and deduced its molecular weight. Then, we performed LC-MS/MS analysis to identify the different proteins in the venom. We also studied the antimicrobial activity of the venom and conducted protein–protein interaction studies to determine possible interactions between the venom neurotoxin and human potassium/sodium channels.

## 2. Results

### 2.1. Morphological Identification

**Figure A1:**
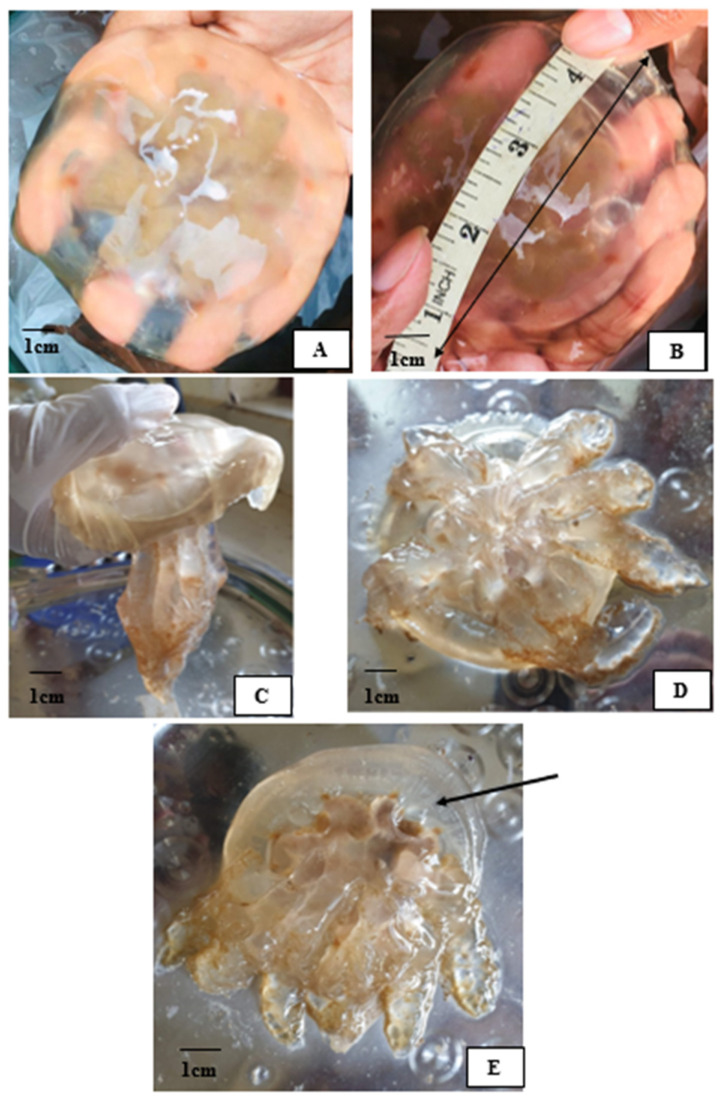
Morphology of jellyfish specimens collected from Beruwala coastal waters. (**A**) The exumbrella; (**B**) exumbrella diameter (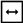); (**C**) side view; (**D**) oral arms; (**E**) subumbrella region (arrow).

The bell of this species was 115 mm in diameter and 215 mm in circumference. The bell was hemispherical to somewhat flattened with a thick but rather smooth surface without any marginal tentacles. Oral arms (8.76 cm) were shorter than the diameter of the bell. The animal was of uniform creamy white color. The large bell was transparent with brownish spots near the margin. Eight ribbon-like oral arms with no appendages were observed. Gonads were visible through the transparent exumbrella surface and were green and brown in color (Figure A1). Based on these morphological measurements and descriptions, it can be concluded that the collected specimens belonged to the genus *Catostylus*. 

### 2.2. Phylogenetic Analysis

**Figure 1:**
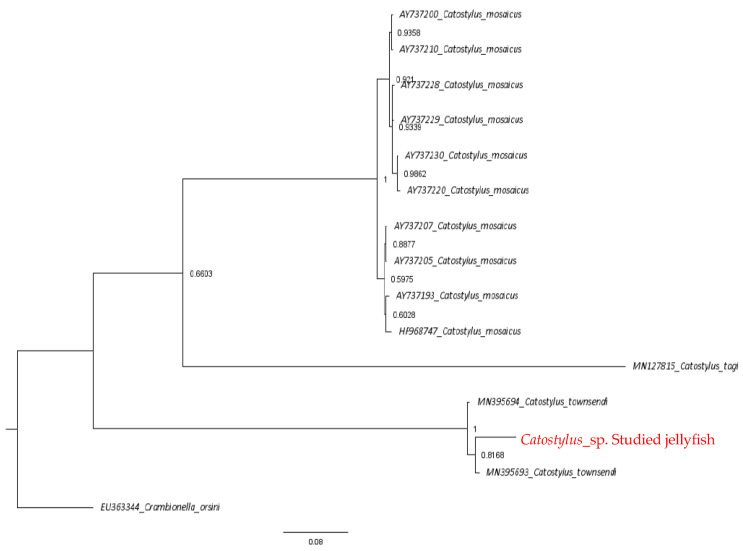
Bayesian tree of concatenated sequences (*COI* and *ITS1*). The studied jellyfish is highlighted in red. Numbers at the nodes indicate posterior probability values.

Trees estimated from both analysis methods confirmed that the studied jellyfish is *Catostylus* sp. Both trees were rooted to the outgroup *Crambionella orsini* and *Crambionella helmbiru*. Species in the genus *Catostylus* showed more than 90% of the highest sequence similarity to the studied jellyfish in the current study, and the E values were zero. Sequence analysis revealed that the sequence similarity to the species level was lower than the genus level. The studied jellyfish sequence was 97.75% to 98.11%, similar to *Catostylus townsendi.* In contrast, it was lower (84–86%) with *Catostylus mosaicus*. The *COI* gene sequence generated in the study was aligned with 25 reference sequences, including the outgroup species sequence obtained from GenBank. The final sequence alignment length was 645 bp. The alignment contained 182 variable sites, of which 168 were parsimony informative sites, and 14 were singleton sites. All the taxa showed similar nucleotide composition, with an average of thymine (T) = 35.1%, cytosine (C) = 18.8%, adenine (A) = 26.4% and guanine (G) = 19.7%. The final alignment was tested for the best-fit evolutionary model. The model of evolution search conducted by PartitionFinderV1.1.1 selected F81 for subsets 1 and 2 and HKY+I for subset 3 as the most suitable evolutionary models for the final alignment to construct the Bayesian inference. The resulting Bayesian tree is depicted in Figure 1.

Phylogenetic analyses of concatenated gene sequences revealed that partial *COI* and *ITS1* show evidence that the studied jellyfish is a *Catostylus* sp. with high bootstrap values. Two clades, *Catostylus mosaicus* and *Catostylus townsendi*, were distinct. *Catostylus tagi* was a sister taxon to *Catostylus mosaicus*, and it further separated into two sub-clades of *C. mosaicus*. *C. townsendi* also resolved into two clades while clustering with the studied jellyfish. Thus, the identification of the studied *Catostylus* sp. was confirmed by its clustering with *Catostylus townsendi* with high support values at the species node.

### 2.3. Analysis of Nematocysts

**Figure 2:**
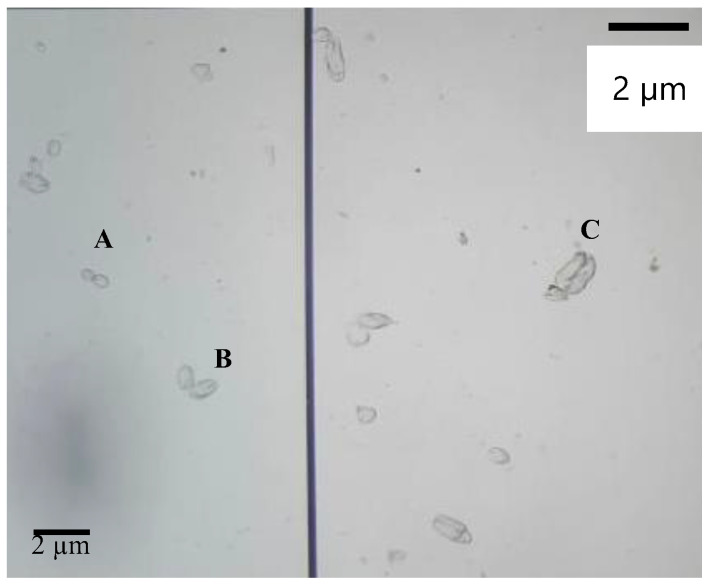
Light micrograph of nematocysts after autolysis at 10 × 10 magnification. (A) 4–6 µm, (B) 7–9 µm, (C) 14–16 µm.

Nematocyst-bearing tissues and isolated nematocysts were microscopically examined at different times during venom extraction. When viewed under a light microscope, the nematocysts appeared to exist in the ridges of the tissue. The isolated nematocysts were in three distinct size groups (Figure 2) of 4–6, 7–9, and 14–16 μm. The localization and size ranges of nematocysts are consistent with the anatomy of the cnidarian venom system and contribute to the existing body of knowledge by confirming the presence of these size groups in *Catostylus* sp. 

### 2.4. Determination of Protein Content

**Figure 3:**
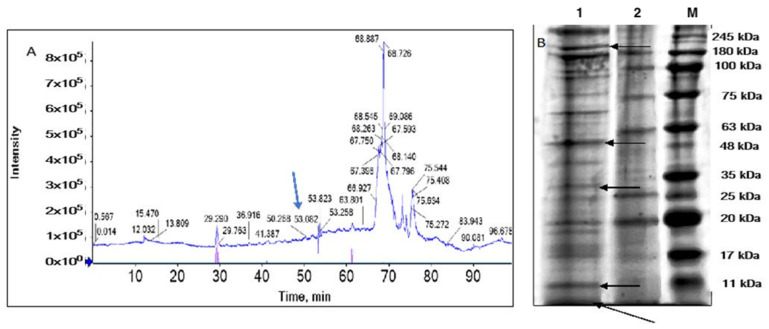
*Catostylus* sp. venom proteins. (**A**) The spectral counts for proteins from toxins using LC-MS analysis and (**B**) SDS-PAGE protein profile of venom samples. **M**—Protein marker, **1**: 25 μg/mL, and **2**: 12.5 μg/mL of 20 µL of crude venom samples. Black arrows indicate venom proteins, while blue arrows indicate the potassium channel toxin alpha-KTx 1.13.

**Figure A2:**
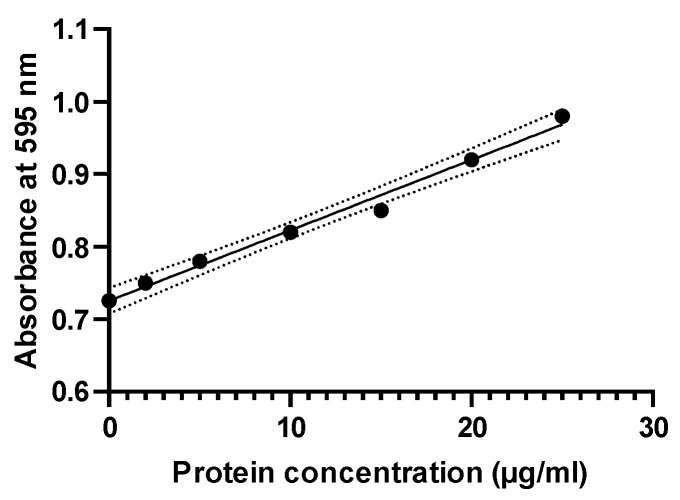
Bovine serum albumin (BSA) microtiter Bradford assay standard curve. The data are fit with the equation y = 0.0097x + 0.72, and the coefficient of determination (R^2^) for this regression model was 0.98, indicating a strong linear relationship between protein concentration and absorbance.

Proteins with a concentration of 12.56 + 0.07 μg/mL were recorded in the venom extract (Figure A2) prepared by sonicating 200 mg of nematocysts in 1200 mL of phosphate buffer. It indicated that nematocysts of this *Catostylus* sp. contain relatively high amounts of venom proteins. The crude extract of the venom fractioned by the C18 nano-LC column (Figure 3A) exhibited a prominent high peak at 68.726 min after entering the sample. From the collected fractions, 13 proteins were identified from the *Catostylus* sp. nematocyst venom extract. The spectrum exhibited seven major peaks with native molecular masses ranging from 6 to 245 kDa (Figure 3B), confirmed by the SDS-PAGE analysis of the toxin proteins in *Catostylus* sp. venom. 

The venom proteins included potassium channel toxin alpha-KTx 1.13 and 12.5, toxin BmKaTx10, turripeptide VIII-01, basic phospholipase A_2_ sistruxin B and OS_2_, phospholipase A_1_, fragments of snake venom serine protease pictobin, thrombin-like enzyme elegaxobin-1, CrTX-A, small cysteine-rich protein 1 2, venom factor and putative antimicrobial peptide 7848, which belonged to the significant toxin families of potassium channel inhibitors, sodium channel inhibitors, phospholipases, serine proteases, pore-forming toxins, venom allergens, proteinase inhibitors and antimicrobial peptides (Table 1). Potassium channel toxin alpha-KTx 1.13 was abundant, showing 53.9% coverage of identified toxin proteins, followed by potassium channel toxin alpha-KTx 12.5 at 15%. Therefore, the major toxin group of *Catostylus* sp. was neurotoxins, including the toxins PLA2, cysteine-rich proteins, toxin BmKaTx10, and tripeptides in this group. 

### 2.5. Docking Analysis

**Figure 4:**
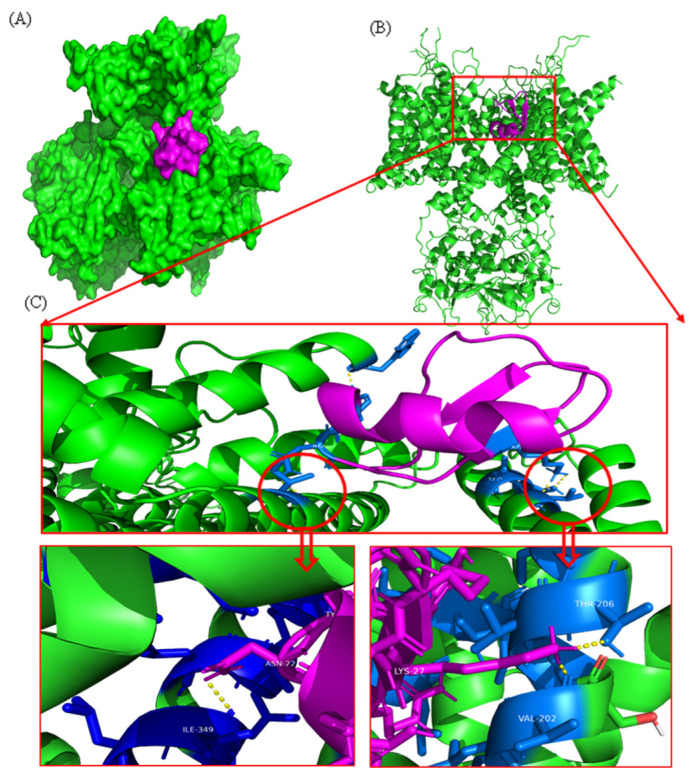
Three-dimensional toxin–potassium complex model from the ClusPro method. (**A**) Surface model. (**B**) Cartoon model. (**C**) Visualization of polar–polar interaction between the toxin–potassium channel complex. (Human voltage-gated potassium channel Kv3.1 (PDB ID-7PHH) is displayed in green, and the potassium channel toxin alpha-KTx 1.13 (UniProtKB—P59944) is displayed in purple, and active residues of the potassium channel are displayed in blue sticks).

The top-ranked docked model of the toxin–potassium channel complex is shown in Figure 4. The weighted score (balanced model) obtained from the ClusPro 2.0 tool for this complex was −941.8. The PRODIGY binding affinity resulted in −17.9 kcal/mol. According to the results of PRODIGY, this complex consists of four polar–polar interactions between the two proteins (Figure 4). 

**Figure 5:**
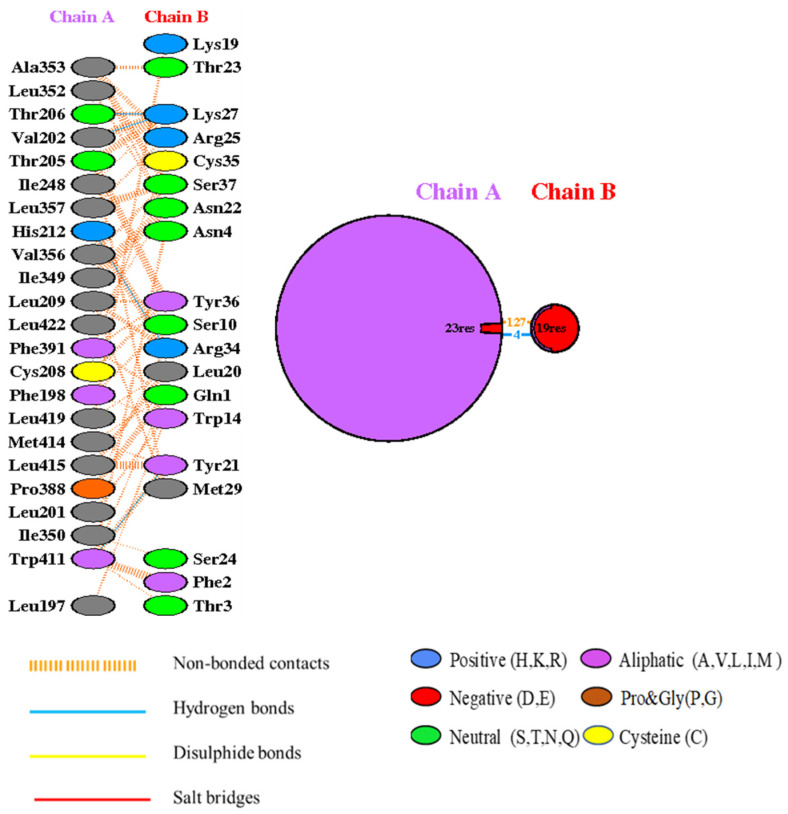
The 2D interaction representation, including hydrogen bonds, salt bridges, and nonbonded interactions between human voltage-gated potassium channel Kv3.1 and potassium channel toxin alpha-KTx 1.13. (Chain A represents the human voltage-gated the potassium channel, and Chain B represents the potassium channel toxin).

Interaction analysis conducted by the PDB-sum webserver shows that there are four hydrogen bonds and 127 non-bonded interactions between the attachment surface of the toxin and one chain of the potassium channel. When forming a polar–polar interaction between the two proteins, the Trp411, His212, Val202, and Thr206 residues act as active residues of the potassium channel. The active residues of the protein toxin are Tyr21, Arg34, and Lys27 (Figure 5).

### 2.6. Antibacterial and Cytotoxicity Activities

The antimicrobial results obtained are depicted in Table 2. According to the results, the venom extract exhibited antibacterial activity against the gram-negative bacterium *Pseudomonas aeruginosa* with a 12 mm inhibition diameter compared to the control (19 mm). However, the venom extract did not exhibit any cytotoxicity activity as determined by the MTT assay.

## 3. Discussion

Jellyfish produce an array of toxic proteins stored and delivered by their nematocysts. Although these toxins are primarily used for prey capture and possibly predator deterrence, their painful and destructive effects on envenomated people make them therapeutically relevant. Despite several decades of biochemical and toxicological studies of scyphozoans, few publications have dealt with the jellyfish *Catostylus* sp. nematocysts venom. Here, we focused on extracting crude venom from *Catostylus* sp. nematocysts and identifying toxin proteins by analyzing the venom directly. A comprehensive examination of the venom was conducted to deepen the understanding of the potential toxin components underlying the significant toxic effects of *Catostylus* sp. venom.

The morphological differences among *Catostylus* species were not apparent due to the lack of fixed morphological characters. Some *Catostylus* species, especially *Catostylus tripterous* and *Catostylus viridescent*, have been considered doubtful [15]. Previously, it was considered that *Catostylus cruciatus* and *Lychnorhiza lucerne* were synonymous species [15]. However, later, it was confirmed that they belonged to the same species, *C. cruciates* [16], which led to the revision of the genus *Lychnorhiza* [17]. Similarly, *Acromitus flagellates* was often misidentified as *Catostylus mosaics* [18]. Hence, there is an urgent need to revise morphological identification using molecular data to overcome taxonomic disparities.

The comparison of characters between the studied *Catostylus* sp. and *Catostylus townsendi* highlighted that *Catostylus townsendi* possesses some peculiarities not shared with the studied jellyfish, such as highly dichotomous oral arms and conspicuous purple-brown spots on the exumbrella [11]. However, other morphological features, including a hemispherical-shaped exumbrella with an average diameter of 10 cm and slightly shorter oral arms than the exumbrella diameter, were alike in both the studied *Catostylus* sp. and *Catostylus townsendi* [19]. Nonetheless, *Catostylus townsendi* was the closest relative of the studied *Catostylus* sp., and these molecular data confirmed that the studied jellyfish is a *Catostylus townsendi.* Genetic distances also support the relationships observed in the phylogenetic tree. The phylogenetic tree, however, unexpectedly highlights that *Catostylus townsendi* fell into two clades. The two clades might be similar because *Catostylust townsend* is polyphyletic, or there is no morphological divergence of these taxa. However, a genetic difference was observed between the two. 

The lack of available reference gene sequences on the studied species and previous taxonomic studies of the jellyfish *Catostylus townsendi* were common problems in species identification. Thus, future analysis of other conserved genomic regions in both mtDNA and nuclear DNA would benefit the studies of *Catostylus townsendi*.

SDS-PAGE electrophoretic profiling of *Catostylus* sp. crude venom revealed many protein bands of varying molecular weights with a clear separation of high molecular weight proteins. In support, previous studies by Wiltshire et al. [20] detected four major protein bands and a few minor protein bands in the venom extract of *Catostylus mosaicus.* Three bands were greater than 106 kDa, and the smallest was between 106 and 80 kDa. The present study’s molecular weight estimations and imaging analysis showed significantly higher protein bands than those of *Catostylus mosaicus*.

The LC-MS/MS analysis revealed that the crude venom extract of *Catostylus* sp. consists of a complex mixture of proteins and peptides. The identified proteins in the studied *Catostylus* sp. venom supported that proteases, lipases, neurotoxins, and protease inhibitors are common to almost all cnidarians [21]. The prominent toxin group in the studied jellyfish was low molecular weight neurotoxins. Similar results were obtained by Yue et al. [22] with *N. nomurai*. Most protein bands in the protein profile correlated well with the identified toxic proteins except for protein bands with molecular weights of ~75, ~100, and ~245 kDa. These findings pointed to the ubiquity of nontoxic proteins, such as components of the nematocyst capsules, in the venom extract. It was the major disadvantage of mechanical disruption of the isolated nematocysts. Therefore, it was difficult to estimate the diversity of nematocyst venom proteins from SDS-PAGE alone because the extent of tissue contamination might prevent protein migration through the gel [21].

Noticeably, toxins targeting voltage-gated potassium channels and sodium channels exhibited high proteome coverage in *Catostylus* sp. venom. This suggested that these toxins highly contribute to the toxic activities of *Catostylus* sp.; thus, the nervous system is the main target of this jellyfish. In support, Zare et al. [23] discovered the neurotoxic activity of crude venom of *Catostylus mosaicus*, which was powerful enough to inhibit muscle contraction. Based on these findings, the present study proposes that *Catostylus* sp. possesses neurotoxic activity. Here, we confirmed the neurotoxin activity using ligand screening with human voltage-gated potassium channel Kv3.1 and potassium channel toxin alpha. We observed four potential active site interactions between the human Kv protein and the protein toxin with a docking score of −941.8 kcal/mol, showing a powerful interaction [24,25].

Similarly, 2D structure analysis of the active sites showed the involvement of van der Waals and hydrogen bonding interactions between human potassium channel residues of Trp411, His212, Val202, and Thr206 with the protein toxin Tyr21, Arg34, and Lys27 residues. While molecular docking is an effective approach for identifying potential interactions between jellyfish toxins and human potassium channels, its limitations must be recognized. Combining docking with other computational tools, such as molecular dynamics simulations, and testing predictions with experimental data can help to address these limitations and provide a more complete knowledge of toxin–channel interactions. Further clarification, such as molecular dynamics simulations, has to be conducted for more accurate binding data and complex stability prediction. Ion channels play a significant role in regulating the permeability of neurolemma to generate electrical signals that propagate information across the body. By modifying channels, cnidarian neurotoxins could prolong the action potential of excitable and non-excitable membranes in sensory neurons and cardiac and skeletal muscle cells [26]. Hence, neurotoxins that act as voltage-gated potassium ion channel (Kv) blockers could be promising targets for neurodegenerative disease therapeutics [27].

Both PLA_1_ and PLA_2_ family proteins were discovered in *Catostylus* sp. venom. Moreover, the present study also reports two PLA_2_ family proteins with virtually comparable coverage levels. Azila et al. [28] proposed that the hemolytic activity in the extract from the oral arms is associated with PLA_2_. Therefore, the present results supported the previous observations of *Catostylus* sp. having phospholipase activity on its host. However, this is the first report of PLA_1_ in *Catostylus* sp. venom. A similar cysteine-rich protein in the studied species was previously found in *Catostylus mosaics* [21]. *Catostylus* sp. shared gene sequence similarities to cysteine-rich proteins found in *Malo Kingi*, *Nematostella vectensis*, and snake venom. Therefore, it confirmed that this toxin protein represents fragments of the *Catostylus* envenoming system.

Even though the hemolytic, edema- and hemorrhage-inducing activities of *Catostylus mosaics* [28] and the hemolytic activity of *Catostylus tagi* [29] have been reported in the literature, no study has identified proteins that induced these activities, except for PLAs. Experimental studies using other jellyfish venom have demonstrated that serine proteases, such as toxin CrTX-A, could cause hemolysis, cytotoxicity, dermo-necrosis, inflammation, and pain [30]. Therefore, these homologous toxins in the venom of *Catostylus* sp. could also cause similar symptoms once stung. 

The UniProt search confirmed that the antimicrobial peptide observed in the *Catostylus* sp. was homologous to putative antimicrobial peptides from *Urodacus yushchenko*. Although a variety of antimicrobial peptides have been discovered in multiple cnidarians, only three jellyfish species, the cubozoan *Carybdea marsupialis* [31], the scyphozoan *Aurelia aurita* mesoglea [32], and *Chrysaora quinquecirrha* [33], had shown antimicrobial activity. Considering the results, the peptide 7848 protein could be classified as another antimicrobial peptide discovered in scyphozoan toxins. We confirmed the antimicrobial activity of peptide 7848 against the gram-negative *Klebsiella pneumonia*. Oppong-Danquah et al. [4] demonstrated that *P. periphylla* exhibited antibacterial activity against gram-negative bacteria. Hence, peptide 7848 could be an initiative for a novel antimicrobial compound. In summary, this is a detailed description of the venom protein composition of the jellyfish *Catostylus* sp.

## 4. Materials and Methods

### 4.1. Materials

All chemicals used in the following venom extraction were of analytical grade and were purchased from Sigma Chemical Company (Pvt) Ltd. (St. Louis, MO, USA) unless stated otherwise. All reagents were prepared according to standard methods. The SDS-PAGE kit was purchased from HiMedia Laboratories (Pvt) Ltd. (Mumbai, India). All chemicals used in the following molecular analysis were of molecular biological grade and were purchased from Sigma Chemical Company Ltd. (St. Louis, MO, USA) unless stated otherwise. The QIAGEN DNeasy blood and tissue kit was purchased from Microtech Biological (Pvt) Ltd. (Hilden, Germany). All deoxynucleotide triphosphates (dNTPs) and Taq DNA polymerase were purchased from Promega Corporation (Madison, WI, USA). Custom made oligonucleotides (primers) were purchased from Integrated DNA Technologies, Inc. (Coralville, IA, USA). The appropriate precautionary measures were adopted to ensure the purity of the products utilized.

### 4.2. Sample Collection and Identification

Jellyfish were collected using traditional beach seines from Beruwala (6028′44.0″ N 79058′59.2″ E), Western Province, Sri Lanka. The species was identified using morphological and molecular approaches. Morphological identification was based on taxonomic descriptions proposed by Kramp [19] and Kitamura and Omori [34].

### 4.3. DNA Extraction and Sequencing

Molecular identification of jellyfish species using phylogenetic approaches was based on DNA sequencing of the mitochondrial cytochrome oxidase I (*COI*) and internal transcribed spacer 1 (*ITS1*) regions. The bell margins were excised manually from living specimens and were snap-frozen in liquid nitrogen until transport to the laboratory and stored at −80 °C until use. DNA was extracted using a QIAGEN DNeasy blood and tissue kit following the manufacturer’s protocol. Polymerase chain reaction (PCR) was used to amplify the 710 bp fragment of the *COI* gene and the 686 bp fragment of the *ITS1* gene to be utilized in sequencing (BIOER. Life ECO Thermal Cycler). LCO1490 was used as the forward primer, and *HCO2198* was used as the reverse primer proposed by Folmer et al. [35] to amplify the *COI* and *ITS1* regions. After the reaction, the PCR products were subjected to 1.5% agarose gel electrophoresis and sent to Macrogen Inc., Seoul, Republic of Korea, for recycling and sequencing.

### 4.4. Phylogenetic Analysis

DNA sequence reads were checked and edited manually using DNA Baser Assembler software (https://www.dnabaser.com/, accessed on 12 December 2023) to remove primer sequences and construct contiguous sequences. The identity of sequences was verified by the Basic Local Alignment Search Tool (BLAST), which searched against GenBank sequences in the National Center for Biotechnology Information (NCBI), USA, for preliminary determination of the jellyfish species [36] Sequences of *Crambionella orsini* were included as outgroup taxa. Multiple sequence alignments and analyses were performed for each gene region using ClustalW in MEGA version 5.2 [37].

The final alignments were tested for the best-fit evolutionary model using PartitionFinder V2.0 (Pathfinder 2). Determined evolutionary models were incorporated into the construction of the Bayesian inference analyses. Bayesian analyses were performed using MrBayes 3.2.7 [38]. Four Markov chains were run for 500,000 generations with a sampling frequency of 100. The deviation of split frequencies was less than 0.01. Twenty-five percent of the initial trees were discarded, and the remainder were used to construct a consensus tree. Uncorrected p genetic distances were obtained for the sequenced specimens using MEGA 5.2 [39].

### 4.5. Nematocysts Isolation and Venom Extraction

Fresh tentacles were cut off from another set of captured live jellyfish to avoid nematocyte mechanical discharge and were stored at −80 °C until further use. Nematocysts were isolated from frozen bell margins according to the procedure by Feng et al. [8]. Excised bell margins were refrigerated with two volumes of filtered and precooled seawater for one to four days. Once a day, the containers were gently swirled, and aliquots were filtered through a fine kitchen sieve. The filtrate was centrifuged (HERMLE Labotechnik: Z 306) at 6000 rpm for 15 min at 4 °C. The sediment was collected and washed three times using filtered seawater. All the final undischarged nematocysts were collected, examined microscopically, and stored at −80 °C until further use.

A weight of 200 mg of frozen nematocysts was resuspended in a volume of 1200 mL of 10 mM phosphate buffer (pH 6) as in a previously published procedure [8]. Crude venom was extracted by sonication (TF 1000) in three periods of the 20 s on dry ice. The extract was then separated from crushed capsules by centrifugation at 15,000 rpm at 4 °C for 20 min. The final solution was stored at −20 °C until further use.

### 4.6. Determination of Protein Concentration

The method described by Bradford [40], with modifications, was used to determine the venom’s protein concentration. Ten μL of the solution was added to the microtiter plate in triplicate, and then 300 μL of Coomassie dye reagent was added to each well. The plate was kept at room temperature for 10 min. The absorbance was measured at 595 nm by a microplate reader (Biobase EL10A, Shandong, China), and the values were compared with bovine serum albumin (BSA) reference standards.

### 4.7. SDS-PAGE Analysis and LC-Ms/MS Analysis

The proteins in the venom extract were analyzed using SDS-PAGE, where 20 µL of the sample was denatured by boiling for 5 min in 5 µL of 5× sample loading buffer. The proteins were separated using a 5% stacking gel and a 12% separating gel and stained with Coomassie Brilliant Blue R-250. The molecular weights were compared with a pre-stained protein ladder, 6–250 kDa (HiMedia, Mumbai, India). For LC-MS/MS analysis [41], the SDS-PAGE gel lane containing the venom protein was decolorized and treated with 10 mM DTT. The samples were digested overnight with 1 μg/μL trypsin in 50 mM triethylammonium bicarbonate (pH 8.5). Then, the samples were centrifuged for 5 min and loaded into a C^18^ column washed with 0.1% formic acid and 4% acetonitrile thrice and eluted twice with 0.1% formic acid and 75% acetonitrile. The re-lucent of the samples was lyophilized and dissolved in 10 μL of mobile phase. Subsequently, the sample was loaded onto the C18 nanotrap column (3C18-CL, 75 μm × 15 cm, Eksigent Technologies, Dublin, CA, USA). Peptides were then separated using an analytical column, followed by a linear gradient method, which was set as follows: 0–1 min, 5% B; 1–60 min, 5–30% B; 60–66 min, 30–80% B; 66–74 min, 80% B; 74–85 min, 80–85% B; 85–100 min, 5% B using mobile phase A (0.1% HCOOH in water) and mobile phase B (0.1% HCOOH in acetonitrile). The final product was analyzed using a mass spectrometer coupled with an ion source of Nanospray Flex. The protein spectra were searched in Tox-Prot https://www.UniProt.org/program/Toxins; accessed on 12 December 2023). Each protein should contain at least one unique peptide, and ion scores > 11 indicate broad homology.

### 4.8. Antibacterial Activity

The antimicrobial sensitivity of jellyfish venom was assessed using the disc diffusion assay against two gram-positive (*Bacillus cereus*: ATCC 11778; *Staphylococcus aureus*: ATCC 25923) and two gram-negative (*Escherichia coli*: ATCC 25922; *Pseudomonas aeruginosa*: ATCC 25853) infectious bacterial strains. Ampicillin was used as the positive control, while phosphate buffer was used as the negative control.

Prepared Mueller–Hinton agar was spread on sterile Petri dishes and allowed to solidify. Bacterial strains were sub-cultured overnight, and the suspension was adjusted to 5 × 10^8^ CFU/mL. Subsequently, bacterial strains were swabbed on agar plates, and sterile filter paper discs of 6 mm diameter were impregnated with different concentrations of the venom extract (250 ppm and 500 ppm), positive control (250 ppm), and negative control [42]. The plates were kept at 37 °C for 24 h, and the inhibitory zones were measured for three replicates.

### 4.9. Cytotoxicity Studies

The cytotoxicity of the venom extract was assessed using the Vero cell line CLL-18 (ATCC) at King Abdullah International Medical Research Center (KAIMRC). In brief, the Vero cell line was grown in DMEM media with 10% FBS and 1% penicillin-streptomycin antibiotic solution. The cells were incubated at 37 °C in a 10% CO_2_ incubator. The cytotoxicity was assessed using 3-(4, 5-dimethyl thiazolyl)-2,5-diphenyl-tetrazolium bromide for Vero cells seeded in 96-well plates at a density of 1 × 10^6^ cells in 100 μL of DMEM media incubated for 48–72 h with different concentrations (20–1000 μg/mL) of the venom extract [43].

### 4.10. Functional Analysis of Neurotoxicity through Molecular Docking

The human voltage-gated potassium channel Kv3.1 crystal structure was downloaded from the RCSB Protein Data Bank (PDB ID-7PHH). The potassium channel structure was analyzed and visualized using PyMOL 2.6 software (Figure 4). The recently predicted AlphaFold 3D structure of potassium channel inhibitor toxin KTx 1.13 (UniProt accession number P59944) was obtained from the UniProt website. A molecular protein–protein docking study was conducted using the ClusPro 2.0 online web server. ClusPro is a standard tool with over 200 citations [43,44]. The PRODIGY web server was used to analyze the binding affinity values of the toxin to the potassium channel complex [25,44]. The toxin and channel protein were observed. LigPlot + v.2.2 generated 2D interaction diagrams. Interaction analysis was conducted using the PDBsum web server [45].

### 4.11. Statistical Analysis

Figures were generated using GraphPad Prism 8.0 (GraphPad Software, San Diego, CA, USA), and tables were constructed in Microsoft Excel 2019 (Microsoft Corp., Redmond, WA, USA). Each experiment was performed with a minimum of three biological replicates (n = 3), and all assays were repeated at least three times to confirm reproducibility. The results are presented as the mean ± standard deviation.

## 5. Conclusions

The research findings provide awareness of the growing repertoire of jellyfish venom, proteomics, and their clinical effects on the skin, nervous system, immune system, and hemostasis. This provides a detailed description of the venom protein composition of the jellyfish *Catostylus* sp., revealing that their venom contains phospholipases, serine proteases, pore-forming toxins, venom allergens, venom factors, antimicrobial peptides, and sodium channel inhibitors, with potassium channel inhibitors being the most abundant. It also confirms that peptides and high molecular weight protein toxins have valuable pharmacological and antibacterial properties. Ligand screening confirmed potent potassium channel inhibitory activity, which could be used as potential therapeutics for neurodegenerative and infectious diseases, underscoring the venom’s potential in drug development.. These findings offer valuable insights into *Catostylus* sp. venom complexity and its diverse biological activities while comparatively providing a basis for further research into toxin diversification and its potential applications in pharmacology and medicine, opening avenues for developing novel therapeutics based on these natural toxins.

## Figures and Tables

**Table 1:** Potential toxin components of venom were identified through similarity searching of LC-MS/MS data relating to peptides isolated following nematocyte proteomic analysis.

Peptide	Accession Number	Molecular wt. (Da)	Organism	Mode of Action
Toxin KTx 12.5	P0CH12	6720	*Lychas mucronatus*	Potassium ion channel inhibitor
Toxin BmKaTx10	Q9NJC5	9374	*Mesobuthus martensii*	Sodium ion channel inhibitor
Turripeptide VIII-01	D5KXH3	16,124	*Gemmula speciosa*	Neurotoxic
Basic phospholipase A_2_ sistruxin B	Q6EER2	15,844	*Sistrurus tergeminus*	Phospholipase A_2_
Phospholipase A_2_	Q45Z47	16,104	*Oxyuranus scutellatus*	Phospholipase A_2_
Phospholipase A_1_	Q9U6W0	33,484	*Polistes annularis*	Phospholipase A_1_
Snake venom serine protease pictobin	U5YCR8	27,783	*Bothrops pictus*	Serine protease
Thrombin-like enzyme elegaxobin1	P84788	25,440	*Protobothrops elegans*	Serine protease
Venom factor	J3S836	184,923	*Crotalus adamanteus*	Proteinase inhibitor
Toxin CrTX-A	Q9GV72	49,392	*Carybdea rastonii*	Pore-forming toxin
Small cysteine-rich protein 1 2	C0H694	8892	*Montipora capitata*	Venom allergen
Putative antimicrobial peptide 7848	L0GCJ6	8854	*Urodacus yaschenkoi*	Antimicrobial

**Table 2:** Diameter of microbial growth inhibition (mm) exhibited by the nematocytes extracted from *Catostylus* sp. against different microbial strains.

Strain	Inhibitory Diameter (mm)
*Bacillus cereus*	-
*Staphylococcus aureus*	-
*Klebsiella pneumonia*	12 ± 0.06
*Pseudomonas aeruginosa*	-
*Escherichia coli*	-
Ampicillin (standard drug)	19 ± 0.07

## Data Availability

All the data needed to support the conclusions of this study are presented in the article and Appendix A. Raw data is available upon reasonable request.
